# Inducible clindamycin resistance and *erm* genes in *Staphylococcus aureus* in school children in Kathmandu, Nepal

**DOI:** 10.2144/fsoa-2020-0092

**Published:** 2020-10-20

**Authors:** Roshan Timsina, Upasana Shrestha, Anjana Singh, Bivek Timalsina

**Affiliations:** 1 Central Department of Microbiology, Tribhuvan University, Kathmandu, Nepal; 2 Center for Health & Disease Studies-Nepal (CHDS), Shankhamul, Kathmandu, Nepal

**Keywords:** antibiotic resistance, D-test, *erm*, erythromycin, inducible clindamycin resistance, methicillin-resistant *Staphylococcus aureus*, Nepal

## Abstract

**Aim:**

Resistance to methicillin and Macrolide–Lincosamide and Streptogramins B and their association with *erm* genes in *Staphylococcus aureus* are unknown in Nepal.

**Materials & methods:**

Nonduplicate nasal swabs from 160 school children were collected from April to September 2018 and processed using standard microbiological procedures.

**Results:**

Out of 160 samples, 64 (40%) were *S. aureus* in which 17 (26.6%) were methicillin-resistance *Staphylococcus aureus* (MRSA). D-test identified 15 (23.4%) as inducible clindamycin-resistant, which were more prevalent in MRSA (76.4%) than methicillin-sensitive *S. aureus* (MSSA; 4.2%). 18.7% of isolates harbored the *ermC* gene followed by *ermA* (15.6%) and *ermB* (3.1%), and were more in MRSA than MSSA.

**Conclusion:**

To prevent treatment failure by inducible resistance, D-test must be performed on erythromycin-resistant and/or clindamycin-sensitive isolates.

## Lay abstract

With the increased prevalence of methicillin-resistant *S. aureus* in hospital- and community-acquired infections, there has been an upsurge in resistance toward Macrolide–Lincosamide–Streptogramin type B antibiotics. This has rendered therapy difficult, thereby increasing morbidity, length of hospital-stay and cost of treatment. Therefore, the correct identification and reporting of *S. aureus* isolates and their susceptibility patterns, more specifically, toward methicillin, clindamycin and erythromycin is very crucial.

*Staphylococcus aureus*, especially methicillin-resistant *S. aureus* (MRSA) was frequently isolated and identified to be the cause of nosocomial and community-acquired infections [1]. Previously, *S. aureus* was sensitive to glycopeptides such as vancomycin and teicoplanin but during recent years these organisms have developed resistance to these antibiotics, which has encouraged physicians to prescribe another family of antibiotics as an alternative; macrolide–lincosamide–streptogramin type B (MLS_B_) [2,3].

Both MRSA and methicillin-sensitive *S. aureus* (MSSA) can be treated by clindamycin, an MLS_B_ antibiotic which is of low cost, and has fewer side effects and high bioavailability in both oral and parenteral forms [4]. Although they are chemically different, the MLS_B_ group exhibits similar inhibitory effects in bacterial protein synthesis [3,5]. There are several mechanisms to MLS_B_ resistance, which include macrolide efflux pump, target site modification and enzymatic antibiotic inactivation [3,6]. Among them target site modification is predominant, which is mediated by the *erm* gene [3,5]. Four genes, namely *ermA*, *ermB*, *ermC* and *ermF*, are frequently associated with resistance to MLS_B_ [7–10]. These genes produce methylase, an enzyme that modifies the ribosomal target site preventing binding of the antibiotic and leading to constitutive and inducible resistance [8,11]. Inducible resistance is developed when a strong inducer of the methylase enzyme, erythromycin, is present. Such an isolate is susceptible to clindamycin but resistant to erythromycin. D-zone effect or erythromycin induction of clindamycin resistance using the disk-diffusion method provides proof of this statement [12]. Therefore, it is very crucial to identify actual MLS_B_ resistance for prescribing appropriate therapy in infected patients [13]. If the patient is prescribed with clindamycin without the proper identification of MLS_B_ resistance, it can lead to treatment failure [14].

We found multiple studies reporting variable rates of inducible clindamycin resistance in different places [2,15–22]. Moreover, prevalence rates of different *erm* genes are still understudied in Nepal. Therefore, the aim of this study is to give an exact picture of what the prevalence of these genes are in the Nepal community. This will provide a proper direction for future researchers as well as prevent health professional from prescribing antibiotics relating to this condition.

## Materials & methods

### Materials

All the microbiological media and antibiotic discs were purchased from HiMedia Pvt. Ltd., Co., Mumbai, India. Other chemicals were purchased as primer (integrated DNA technology), Master mix (Takara Bio Inc., Nojihigashi, Japan), DNA Ladder (GeneDirex, Inc., MD, USA), Good view nucleic acid stain- HGV II (SBS Genetech Co., Ltd, Beijing, China), agarose (GeneDireX, Inc.).

### Sample collection

After the approval from Nepal Health Research Council, National Ethical Guidelines for Health Research in Nepal (reg. no. 195/2O18), a prospective cross-sectional study was conducted over 6 months of period to isolate *S. aureus* from nasal samples collected from students of two different schools of Kathmandu, namely Kirtipur Secondary and Mangal Secondary School. The informed consent was taken from guardian of students before sample collection. Only those participants (school children) who were not taking any medications were included in the study. Nasal swabs were collected by inserting a sterile moistened cotton swabs (HiMedia) into each nostril and transferred to the laboratory keeping in transport media. The laboratory tests were conducted from April–September 2018 in the Microbiology lab of Center for Health and Disease Studies, Nepal. A total of nonduplicate 160 samples were analyzed in the study.

### Bacterial isolation & identification

The specimens collected were inoculated in mannitol salt agar (MSA), blood agar (BA) and incubated at 37°C aerobically for 24 h. Beta hemolytic colonies on blood agar and typical mannitol fermenting colonies in MSA were observed. Pin-point-sized colonies on blood agar with diameter of 2–3 mm were indicative of *S. aureus*. Gram stain, catalase, oxidase, O–F and coagulase (free and bound) test were performed for further identification using a standard microbiological techniques [23].

### Antibiotic susceptibility testing

Antimicrobial susceptibility was studied by the Kirby–Bauer disk diffusion method on a Mueller–Hinton agar plate (12-cm diameter), following Clinical Laboratory Standard Institute (CLSI) guidelines [1,19]. The tested antimicrobial agents were: penicillin (10 U), cefoxitin (30 μg), gentamicin (10 μg), erythromycin (15 μg), clindamycin (2 μg) and ciprofloxacin (5 μg). Cefoxitin (30 μg) was used for the detection of methicillin resistance. Erythromycin (15 μg) and clindamycin (2 μg) discs placed 15 mm apart were used for detection of inducible clindamycin resistance as per recommended CLSI guidelines [23,24].

### Detection of methicillin resistance

With cefoxitin disks, isolates with zone of inhibition ≥22 mm in diameter were considered methicillin resistance and those with ≤21 mm were considered as methicillin susceptible.

### Detection of inducible clindamycin resistance

**Figure 1.**
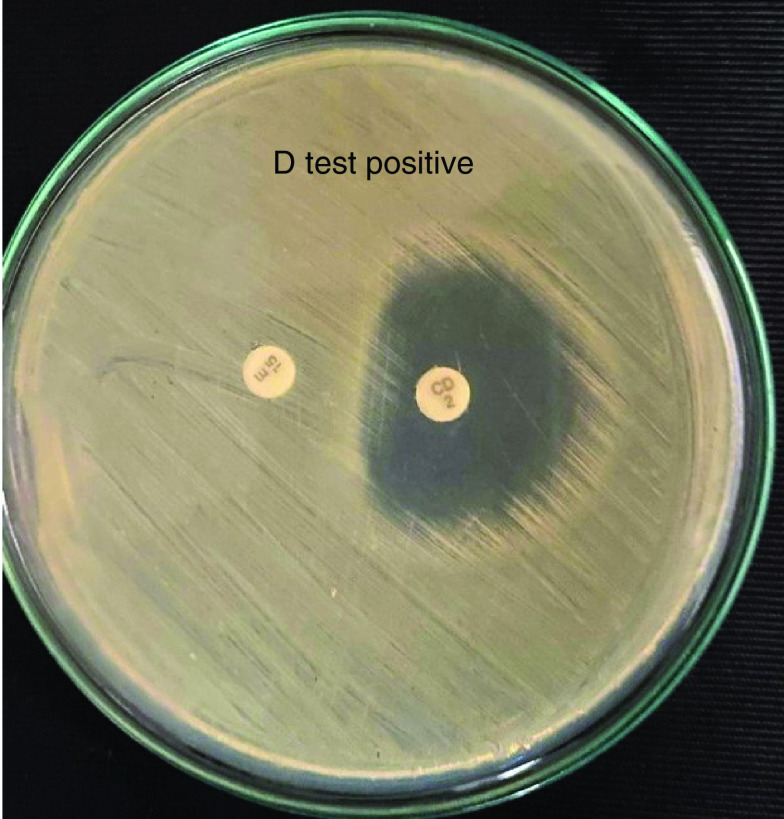
Inducible clindamycin resistant (D-test) positive showing MRSA in MHA media. CD: Clindamycin; E: Erythromycin; MHA: Muller hinton agar; MRSA: Methicillin-resistant *Staphylococcus aureus*.

Formation of a flattening shape of the clindamycin inhibition zone ≥21 mm (D shape) around the erythromycin disk has shown in Figure 1, which indicates that erythromycin has induced clindamycin resistance.

ATCC 25923 strains of *S. aureus* were used to perform quality control. A separate in-house strain that showed inducible clindamycin resistance was also used for quality control.

### DNA extraction

Genomic DNA was extracted from *S. aureus* isolates from QIAamp DNA Mini Kit, Cat No./ID: 51304 (Qiagen). One to two colonies of isolates were taken with nichrome loop, suspended in nutrient broth and incubated for 24 h. The turbidity was checked and DNA was extracted according to the manufacturer’s protocol of QIAamp. The extracted DNA was kept in -20°C until used for PCR.

### Amplification of *ermA*, *ermB* & *ermC* genes

DNA amplification was performed using specific primers for detection of *erm* genes. Primer sequences were designed using Primer-BLAST of NCBI using gene ID 13913675 for *ermA* and 24247827 for *ermC*. Primer sequence for *ermB* was used from research paper [8]. Sequences for primers used for PCR were as follows: *ermA/F*: 5′-AAGCGGTAAACCCCTCTGA-3, *ermA/R*: 5′-TTCGCAAATCCCTTCTCAAC-3 with amplicon size of 190 bp, *ermB/F*: 5′-CATTTAACGACGAAACTGGC-3′, *ermB/R*: 5′-GGAACATCTGTGGTATGGCG-3′ with amplicon size of 142 bp, *ermC/F*: 5′-AATCGTCAATTCCTGCATGT-3′, *ermC/R*: 5′-TAATCGTGGAATACGGGTTTG-3′ with amplicon size of 299 bp. Each reaction was carried out in final volume of 25 μl with Mastermix (12 μl), forward primer (1 μl), reverse primer (1 μl), DNA template (4 μl) and 7 μl nuclease-free water. PCR amplifications were adjusted according to conditions described in previous studies with some modification [12]. Amplification conditions were as follows: initial denaturation (94°C/4 min for *ermA*, 95°C/2 min for *ermB*, 95°C/2 min for *ermC*), denaturation (94°C/30 s for *ermA*, 95°C/30 s for *ermB*, 95°C/30 s for *ermC*), various annealing temperatures (55°C/30 s for *ermA*, 50.2°C/30 s for *ermB*, 52.4°C/30 s for *ermC*) and 72°C/30 s and final extension at 72°/5 min and hold at 4°C for infinity. PCR products were analyzed by separating on 1.8% agarose gel electrophoresis, stained with nucleic acid stain solution and finally visualized in gel documentation system [12]. A reaction containing all materials and nuclease-free water except template DNA was used as negative control. A native isolate harboring *ermA*, *ermB* and *ermC* gene was used as a positive control for *erm* genes. 100 bp ladder from GeneDireX, Inc. was used to identify the size of amplified products.

### Statistical analyses

Microsoft Excel 2016 was used to keep the record of the data. The data were analyzed using SPSS version 25 for Windows. Pearson Chi-square test was used to find the association between the two variables. p-value of less than 0.05 was considered statistically significant.

## Results

### Isolation of *S. aureus* & antibiotic susceptibility pattern

In this study, out of 160 samples processed, 64 samples showed positive culture growth for *S. aureus* in which 17 (26.5%) were MRSA. The antimicrobial sensitivity tests among MRSA showed that all isolates were resistance to penicillin (ten units) and highly sensitive (88.2%) to gentamicin. The results of antibiotic susceptibility testing for other antibiotics are shown in Table 1.

**Table 1.** Result of antibiogram test for methicillin-resistance *Staphylococcus aureus* isolates.

Antibiotics used	Sensitive (%)	Intermediate (%)	Resistant (%)
Gentamycin (10 μg)	16 (88.2)	–	1 (5.8)
Erythromycin (15 μg)	4 (23.5)	3 (17.6)	10 (58.8)
Clindamycin (2 μg)	12 (70.5)	2 (11.7)	3 (17.6)
Penicillin (ten units)	0	0	17 (100)
Ciprofloxacin (5 μg)	6 (35.2)	1 (5.8)	10 (58.8)

### Inducible clindamycin resistance

Inducible clindamycin resistance was seen among 15 (23.4%) of the total isolates. The distribution of iMLS_B_ among MRSA, MSSA and total isolates showed the higher rate of inducible clindamycin resistance in MRSA compared with MSSA (p ≤ 0.05) as shown in Table 2.

**Table 2.** Distribution of inducible macrolide–lincosamide–streptogramin type B among methicillin-resistance *Staphylococcus aureus*, methicillin-sensitive *S. aureus* and total isolates.

Property	MRSA (n = 17)	MSSA (n = 47)	Total isolates (n = 64)
Inducible clindamycin resistance (iMLS_B_)	13 (76.4%)	2 (4.2%)	15 (23.4%)

iMLS_B_: Inducible macrolide–lincosamide–streptogramin type B; MRSA: Methicillin-resistance *Staphylococcus aureus*; MSSA: Methicillin-resistance *S. aureus*.

### Association of *erm* genes in MRSA & MSSA

**Figure 2.**
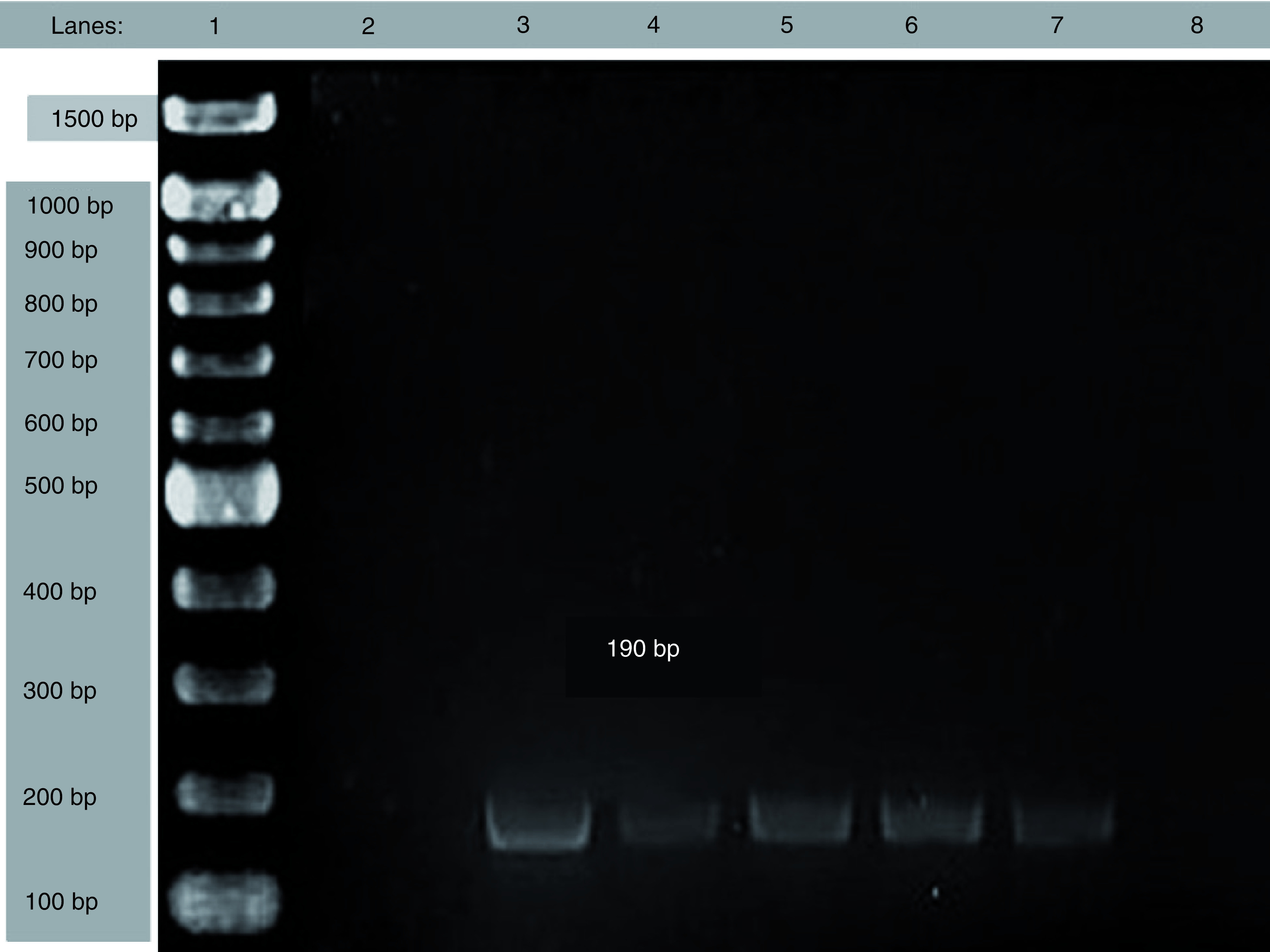
Electrophoresis result of *ermA* gene. Lane 1:DNA marker, Lane 2: negative control; Lane 3: positive control; Lane 4-8: samples and 190 bp for *ermA*.

**Figure 3.**
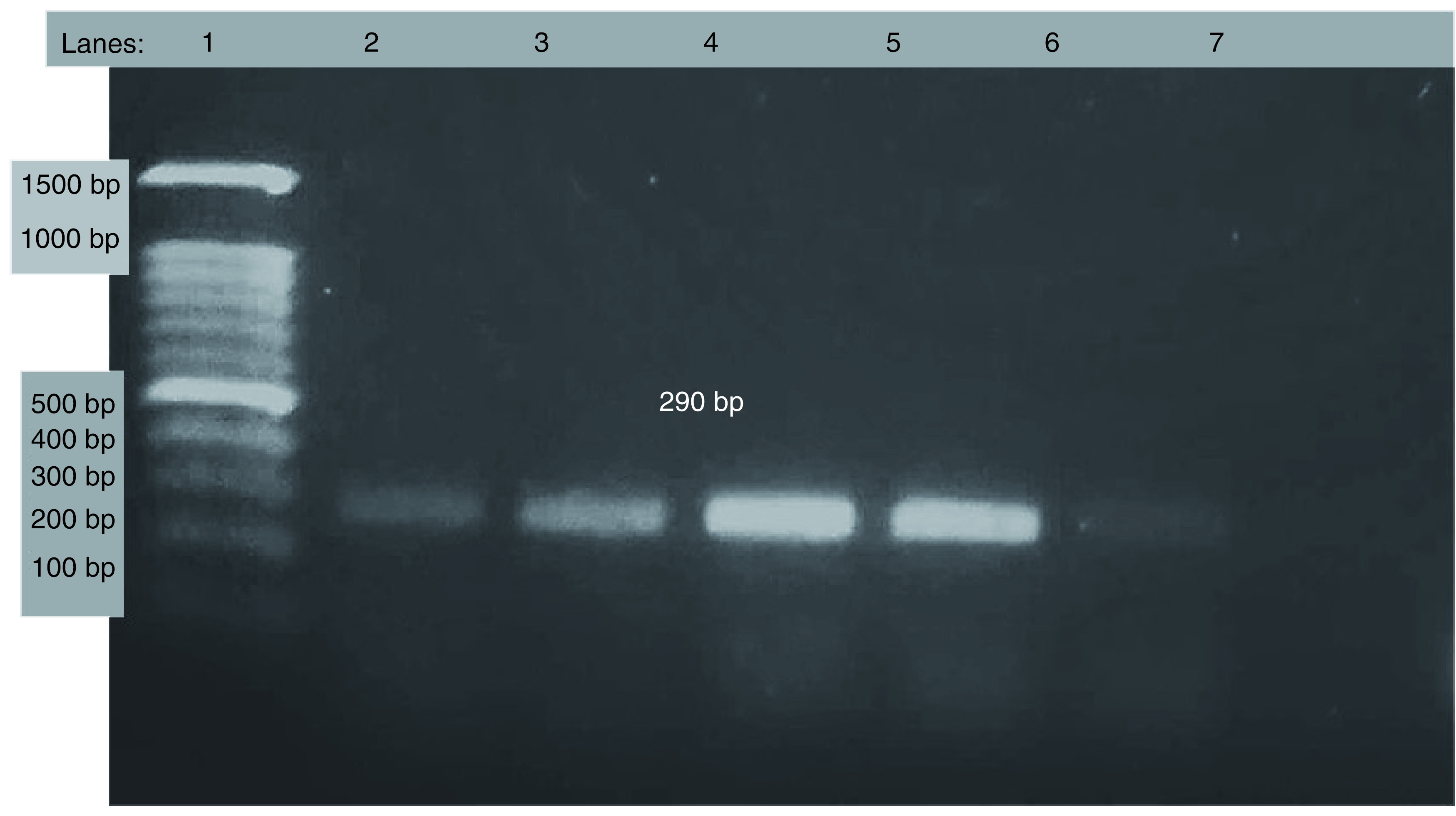
Electrophoresis result of *ermC* gene. Lane 1: DNA marker, Lane 2: positive control; Lane 3-7:samples and 299 bp for *ermC*.

**Figure 4.**
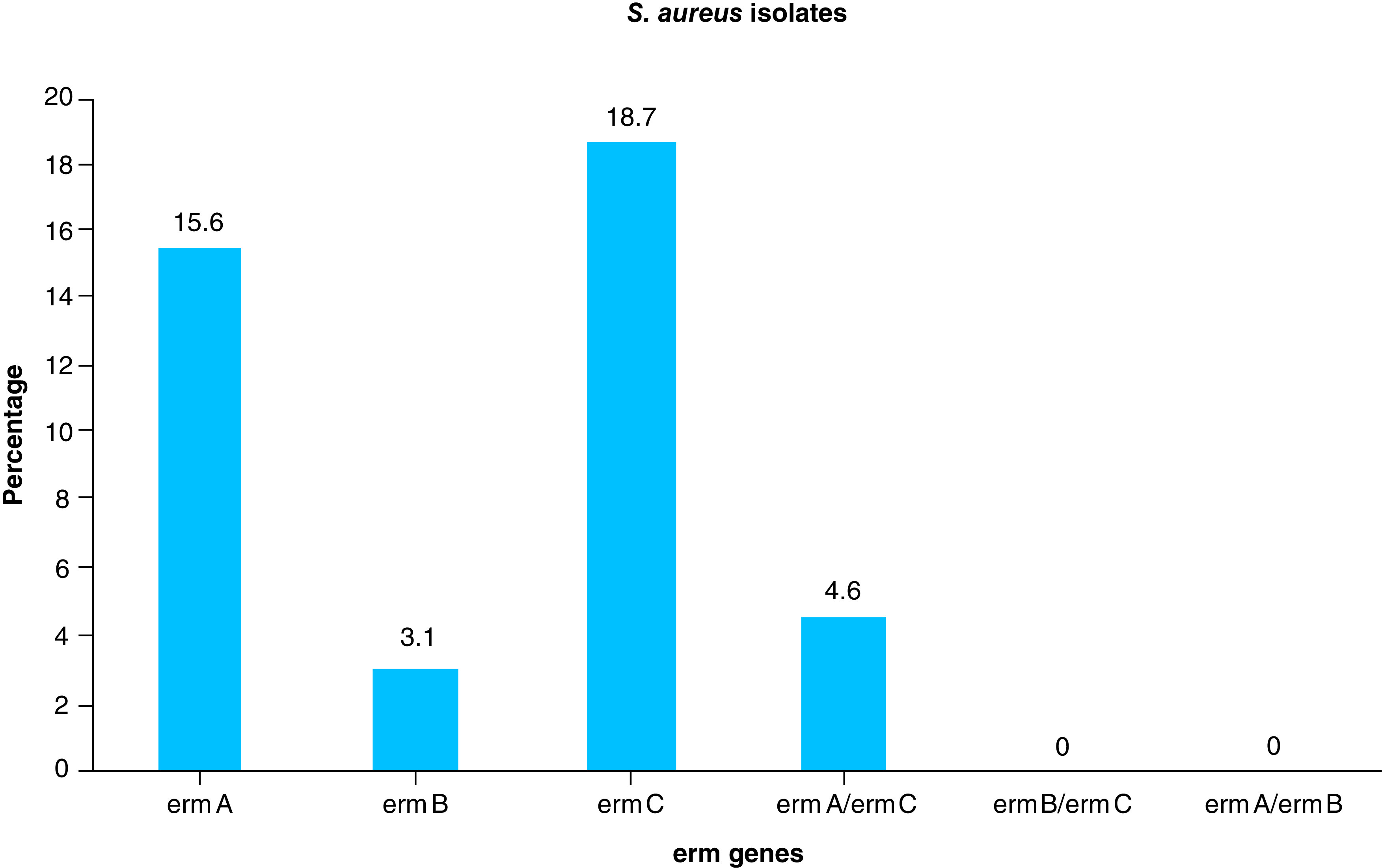
Frequency of *erm* genes in *S. aureus* isolates.

As shown in Table 3 and Figures 2–4, the electrophoresis results of PCR amplicon showed that 15.6% were *ermA* positive, 3.1% were *ermB* positive and 18.7% were *ermC* positive in which three isolates had both *ermA* and *ermC* genes. In this study, the MRSA isolates harbored the *erm* genes in which *ermB* and *ermC* genes were only present in MRSA, whereas few MSSA also had *ermA* genes.

**Table 3.** Result of *erm* genes in isolates according to sensitivity to methicillin.

Genotype	PCR result	Sensitivity to methicillin	
		MRSA (%)	MSSA (%)
*ermA*	Positive	58.8	4.2
	Negative	41.2	95.8
*ermB* [30]	Positive	11.7	0
	Negative	88.3	0
*ermC*	Positive	70.5	0
	Negative	29.5	0

MRSA: Methicillin-resistance *Staphylococcus aureus*; MSSA: Methicillin-resistance *S. aureus*.

## Discussion

*S. aureus* infection is one of the major causes of infection, mainly in low- and middle-income countries and the rate of emergence of antibiotic resistance is quite alarming [25].

We found a significant number of *S. aureus* from school children, which also show resistance to methicillin, erythromycin and clindamycin. It is very important to correctly identify and report *S. aureus* isolates, especially in clinical and diagnostic settings, including whether the isolates are truly susceptible to clindamycin when they are resistant to erythromycin. A simple D-test can be performed in the laboratory, so that inducible clindamycin-resistant isolates can be excluded for clindamycin therapy [26]. Prevalence of *S. aureus* in a community is multifactorial in nature, depending on the geographical location, socioeconomic status, patient age, species of bacteria, inconsistent use of erythromycin and source of the strain (community or nosocomial) [7,27].

The results of our study have shown that the prevalence of inducible clindamycin resistance was 23.4% among all the isolates. As shown in Table 4, several studies conducted in different parts of the world are inconsistent with the prevalence of iMLS_B_ and have previously reported higher variability in prevalence, which ranged from 3.3 to 43% [4,11,13,15,16,21,25,28–33]. Also, the prevalence of iMLS_B_ resistance in MRSA was 76.4%, which is much higher than the prevalence rate previously reported by different studies, which were 12.3–35.9% [4,10,25,29,30,34,35]. This indicates an alarming increase in antibiotic resistance. Although iMLS_B_ resistance in MRSA was higher, it was within the range (4–68%) when compared with data of previously reported studies among MSSA [4,10,25,34,35].

**Table 4.** Comparison of frequencies of *ermA*, *ermB* and *ermC* genes in different studies.

Study (year)	Inducible resistance isolates			*iMLS*_B_ (%)	Ref.
	*ermA* (%)	*ermB* (%)	*ermC* (%)		
Lina *et al.* (1999)	63.2	0.7	25	30.2	[24]
Hamilton-Miller *et al.* (2000)	–	–	–	43	[31]
Fiebelkorn *et al.* (2003)	–	–	–	25.2	[25]
Vivian *et al.* (2010)	29.6	17.1	0.66	3.3	[23]
Moosavian *et al.* (2014)	41.1	Nonprevalent	17.7	32.3	[12]
Aydeniz *et al.* (2015)	81.9	0.9	10.8	27.47	[26]
Havaei *et al.* (2016)	11.11	22.22	44.44	4.18	[22]
Fasihi *et al.* (2016)	11	3.5	20.5	12.5	[11]
Adhikari *et al.* (2017)	–	–	–	11.48	[17]
Khashei *et al.* (2018)	–	–	–	8.6	[9]
Our study	66.67	13.33	73.38	23.4	[30]

Results from our PCR study showed that the prevalence of *ermA*, *ermB* and *ermC* genes were: 15.62, 3.12 and 18.75%, respectively, among inducible clindamycin-resistant isolates. Prevalence of *ermA* gene varied among different studies conducted at different geographic location, which ranged from 11 to 81.9% [11,15,16,28,29,31]. Our study was within this range obtained from those previously conducted. Our study also showed the prevalence of *ermC* gene to be within the range when compared with the rates previously reported by several studies [11,15,16,28,31], which ranged from 0.66 to 44.44%. Due to lack of the pertinent literature in different *erm* genes in Nepal, our study aimed to determine the prevalence of *ermA*, *ermB* and *ermC* genes from *S. aureus* isolates and inducible clindamycin-resistant MRSA isolates. The prevalence of *ermA*, *ermB* [36] and *ermC* genes among iMLS_B_ isolates were 66.67, 13.33 and 73.38%, respectively. The comparison of the prevalence rates of *ermA*, *ermB* and *ermC* genes in different studies are given in Table 2.

## Limitations

Regarding the limitations of our study, it was conducted in two different schools of Kirtipur, Kathmandu, Nepal and so while there may be a chance of us extrapolating our findings and applying it across Kathmandu valley, this may not be representative of the whole country. However, Kathmandu is the most populated city in Nepal. Hence, our findings could well represent the whole country. Due to lack of resources and funding, we were not able to perform genotyping or Staphylococcal cassette chromosome *mec* gene (SSCmec) typing of *S. aureus*. Furthermore, MIC of antibiotics used was not determined.

## Conclusion & recommendations

To conclude, this study identified the significant presence of MRSA in nasal swabs from school children, which clearly emphasizes the importance of sanitation. Furthermore, there is an increase in inducible clindamycin resistance that directly impacts the treatment of the cases. Therefore, this highlights the importance of performing D-test to identify these isolates in detail, which ought to be followed by laboratory in routine identification of *S. aureus*.

## Future perspective

As there is presence of significant numbers of *S. aureus* in Nepalese school children as well as resistance to several antibiotics, more research should be done in this area to identify the pathogens, their virulence factors and antibiotic-resistance patterns in order to mitigate the misuse of antibiotics. Furthermore, characterization of gene sequences and typing of strain will give more insight into the genes involved in resistance development.

**Summary points**

- Methicillin-resistance *Staphylococcus aureus* and inducible clindamycin resistance are emerging as a public health threat, which could lead to increased morbidity and mortality if proper diagnosis and treatment are not done.
- There is a significant association of *ermA*, *ermB*, *ermC* genes and inducible clindamycin resistance, which can be used for rapid diagnosis and treatment.
- Inducible clindamycin resistance was found to be more among methicillin-resistance *S. aureus*.
- Every diagnostic and microbiology laboratories should perform D-test to identify inducible clindamycin resistance if they encounter *S. aureus* isolates sensitive to clindamycin but resistant to erythromycin.
